# Somatic Mutation Patterns in Hemizygous Genomic Regions Unveil Purifying Selection during Tumor Evolution

**DOI:** 10.1371/journal.pgen.1006506

**Published:** 2016-12-27

**Authors:** Jimmy Van den Eynden, Swaraj Basu, Erik Larsson

**Affiliations:** Department of Medical Biochemistry and Cell Biology, Institute of Biomedicine, The Sahlgrenska Academy, University of Gothenburg, Gothenburg, Sweden; National Institute of Environmental Health Sciences, UNITED STATES

## Abstract

Identification of cancer driver genes using somatic mutation patterns indicative of positive selection has become a major goal in cancer genomics. However, cancer cells additionally depend on a large number of genes involved in basic cellular processes. While such genes should in theory be subject to strong purifying (negative) selection against damaging somatic mutations, these patterns have been elusive and purifying selection remains inadequately explored in cancer. Here, we hypothesized that purifying selection should be evident in hemizygous genomic regions, where damaging mutations cannot be compensated for by healthy alleles. Using a 7,781-sample pan-cancer dataset, we first confirmed this in *POLR2A*, an essential gene where hemizygous deletions are known to confer elevated sensitivity to pharmacological suppression. We next used this principle to identify several genes and pathways that show patterns indicative of purifying selection to avoid deleterious mutations. These include the *POLR2A* interacting protein *INTS10* as well as genes involved in mRNA splicing, nonsense-mediated mRNA decay and other RNA processing pathways. Many of these genes belong to large protein complexes, and strong overlaps were observed with recent functional screens for gene essentiality in human cells. Our analysis supports that purifying selection acts to preserve the remaining function of many hemizygously deleted essential genes in tumors, indicating vulnerabilities that might be exploited by future therapeutic strategies.

## Author Summary

Cancer is one of the leading causes of death worldwide. It is caused by the accumulation of genetic changes (i.e. somatic mutations) in cells, often taking many decades. Like the evolution of a species, the evolution of cancer cells is driven by different selection processes. Changes that are advantageous for the fitness of a cancer cell will be retained (positive selection), while disadvantageous characteristics will be removed (purifying, or negative, selection). While positive selection is well studied and has been used to identify new genes that are responsible for the development of cancer, negative selection has remained elusive and is understudied in this context. In this study, we identify genes that show signals indicative of negative selection against damaging somatic mutations. We accomplish this by focusing on genes that have lost one of their two copies, supposedly making cells more vulnerable to damaging mutations in the remaining copy. The identified genes were found to be active in cellular processes which are essential for cancer cell survival and constitute potential therapeutic targets.

## Introduction

Cancer cells evolve by natural selection, which favors advantageous genetic changes (positive selection) and disfavors deleterious changes (negative or purifying selection). Identification of somatic mutation patterns indicative of positive selection has become a major goal in cancer genomics [1,2]. This is motivated by a search for cancer driver genes and pathways that are recurrently activated in tumors but not healthy cells, thus providing possible therapeutic windows. However, similar to healthy cells, cancer cells additionally depend on a large number of basic cellular processes, and it has been demonstrated that some tumors show elevated sensitivity to inhibition of specific essential non-driver genes [3,4]. While these genes should in theory be subject to purifying selection against damaging somatic mutations, these patterns have been elusive in cancer. Contrary to population (germline) genetic variants, which may be subject to strong purifying selection [5], it has even been suggested that somatic tumor evolution is primarily governed by positive selection [6]. This is supported by a lack of depletion of somatic mutations in coding exons [7], as well as the observation that tumors carry many damaging mutations that have evaded purifying selection [8].

However, none of these studies consider the zygosity of the tumors. Indeed, as the vast majority of essential genes have been suggested to be haplosufficient in cancer cells [9], damaging mutations typically cannot be expected to be subject to purifying selection when both copies of the gene are still present, which may explain why purifying selection appears to be limited in somatic mutation data. Tumors are characterized by overall genomic instability, and hemizygous (single-copy) deletions (HeZD) occur frequently throughout the genome. In human cancer cells, it has been shown that such losses may sometimes confer increased sensitivity to shRNA inhibition of the affected genes [10,11]. Similarly, it was recently shown that HeZD of the essential gene *POLR2A*, a subunit of RNA polymerase II, creates a therapeutic vulnerability, as cancer cells carrying only a single functional copy of the gene are more sensitive to inhibition of its protein product [3]. This occurs frequently due to co-deletion with the neighboring tumor suppressor gene *TP53*.

**Fig 1:**
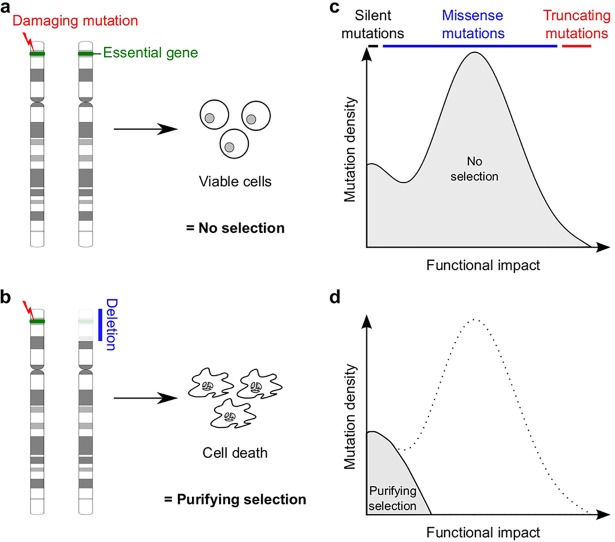
Theoretical concept. **(a-b)** Damaging somatic mutations in HeZD but not CNN (haplosufficient) essential genes are expected to alter cell viability and to be selected against. **(c-d)** This purifying selection will not affect silent and low impact missense mutations, leading to a shift in the overall functional impact when comparing CNN to HeZD tumors.

Here we hypothesize that essential genes like *POLR2A* should display patterns of purifying selection against damaging somatic mutations in the HeZD but not in the copy number neutral (CNN) state, where a complete loss-of-function can be prevented by the healthy allele (Fig 1A and 1B). Purifying selection should then be detectable as a reduction in the number of observed truncating mutations (nonsense mutations and out-of-frame insertions and deletions) and other high impact missense mutations (mutations that have a large effect on protein function) in HeZD tumor samples (Fig 1C and 1D). The main objective is to test whether purifying selection can be detected in essential genes like *POLR2A*, by using a concerted approach exploiting the extensive availability of exome sequencing and copy number data from human tumors.

## Results

### Somatic mutations in *POLR2A* are subject to purifying selection

**Fig 2:**
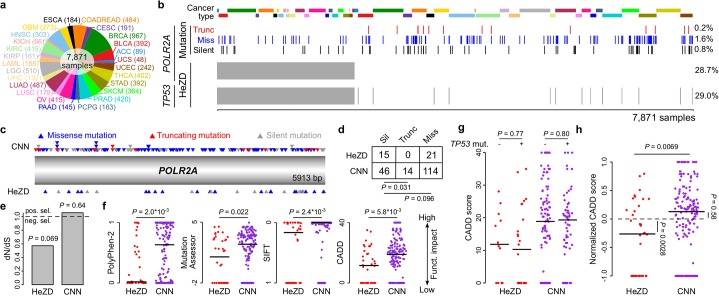
Detection of purifying selection in *POLR2A* in a pan-cancer dataset. **(a)** Twenty-five different tumor types were pooled for analysis. **(b)** Overview of somatic mutations and HeZD in *POLR2A*. HeZD are indicated by grey vertical bars. Silent, missense and truncating mutations are indicated by black, blue and red bars respectively. Frequencies of different events are given on the right of the plot. Cancer types are indicated by color bars on top of the figure. Color legends as in panel **a**. **(c-e)** CDS positions **(c)**, number **(d)** and dN/dS values **(e)** of mutations observed in *POLR2A*. **(f)** The functional impact of all mutations in *POLR2A* is predicted using different functional impact scores as indicated by ordinate labels. **(g)** CADD scores for *POLR2A* mutations in the absence (-) and presence (+) of concomitant *TP53* mutations. **(h)** Normalized CADD scores for *POLR2A* mutations (see Methods and S2 Fig for details). Horizontal lines on plots indicate median values.

To determine whether purifying selection acts on *POLR2A*, we pooled together somatic mutation and copy number data from 7,871 tumor samples, spanning 25 different tumor types, from The Cancer Genome Atlas (TCGA) [12] (Fig 2A). Twenty-nine percent of samples had *TP53*-correlated HeZD of *POLR2A*, and 25% of silent mutations were found in this group (Fig 2B and 2D). However, not a single truncating mutation was observed in the HeZD state, as opposed to 14 in the CNN state (*P* = 0.031, one-sided Fisher’s exact test, comparing the proportion of truncating to silent mutations; Fig 2D). The ratio of non-silent to silent mutations per site (dN/dS), a commonly used metric where values below 1 indicate purifying selection [13,14], was lower than 1 in the HeZD (0.57, *P* = 0.069) but not the CNN state (1.05, *P* = 0.64), consistent with elevated purifying selection in HeZD tumors (Fig 2E). To take into account the variable impact on protein function of missense mutations occurring in *POLR2A*, we calculated several established functional impact scores [15–17]. All scores indicated a lower functional impact in HeZD as compared to CNN samples (*P* = 0.022 or less, Wilcoxon rank sum test; Fig 2F). The Combined Annotation-Dependent Depletion (CADD) score (*P* = 5.8*10^−3^) was preferred for further analysis due to its high accuracy [16]. We next excluded that the reduction in *POLR2A* functional impact score was related to the concomitant loss of function of *TP53*, by showing that this occurs independently of *TP53* mutation status (Fig 2G). While the functional impact difference was most pronounced in breast cancer and metastatic melanoma, no statistical conclusions could be drawn at the level of individual cancers (S1 Fig).

The composition of cancer types differed between copy number states for *POLR2A*, with higher proportions of breast and ovarian cancer in HeZD compared to CNN samples (S2A Fig), which likely reflects increased copy number instability in these cancers [18]. As mutational signatures differ between cancers (as exemplified in S2B Fig) [19,20], we next excluded that this could confound the observed impact scores. For each cancer, we used its specific 96-class mutational signature to simulate CADD impact scores that would be expected in the absence of any selection pressure (S2C Fig; see Methods). Observed CADD scores were then normalized to the median simulated score in each cancer (S2D and S2E Fig) such that a normalized score of zero would indicate no selection. Aggregation of normalized scores across cancers revealed a difference between HeZD and CNN samples that was comparable to the non-normalized approach (-0.26 versus +0.13, respectively, *P* = 0.0069; Fig 2H). This argues against a confounding bias from mutational signatures, and further supports that the difference in impact scores between HeZD and CNN samples was mainly due to purifying selection (negative normalized scores) in HeZD samples (*P* = 0.0028; one-sided Wilcoxon rank sum test), rather than positive selection (positive normalized scores) in CNN samples (*P* = 0.58).

Taken together, our results suggest that purifying selection against high impact mutations is detectable in essential genes like *POLR2A* when subjected to single-allele copy number loss.

### A genome-wide screen of 1,187 loci indicates patterns of purifying selection in multiple genes

**Fig 3:**
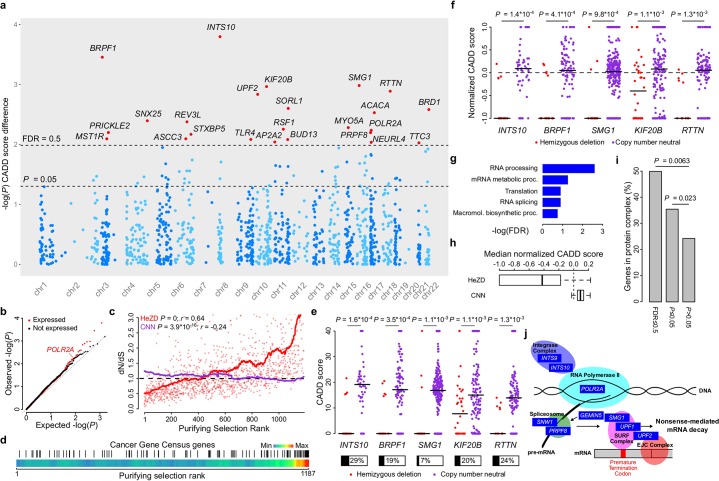
A genome-wide screen unveils patterns indicative of purifying selection. A genome-wide screen was performed on 1,187 genes to detect genes subjected to purifying selection against deleterious somatic mutations. **(a)** Manhattan plot shows all genes that were selected for analysis. Genes at 50% FDR are indicated by red dots and labelled. **(b)** qq-plot shows the difference between expressed genes (as used in the analysis) and non-expressed genes. **(c)** Correlation between the purifying selection rank and dN/dS. Solid lines represent moving averages. For visualization purposes, individual dots of CNN dN/dS values are not shown. Spearman correlation coefficients are shown on top. **(d)** CGC genes are indicated by black vertical bars and shown as a function of purifying selection rank. Color bars indicate the CGC gene density. **(e-f)** Copy number-related CADD scores **(e)** and normalized CADD scores **(f)** from the observed mutations in the top-5 ranked genes. Proportions of samples harboring the deletion are indicated below each plot in panel **e**. **(g)** Top ranked GO biological processes gene set enrichment results. **(h)** Median normalized CADD scores in HeZD compared to CNN samples for the top 76 genes from the purifying selection screen. **(i)** Bar plot shows the proportion of genes with (at 50% FDR or *P*≤0.05) or without (*P*>0.05) signals of purifying selection that are active in protein complexes. **(j)** A high number of genes with signals of purifying selection is involved in RNA-related events and complexes. Horizontal lines on plots indicate median values.

We next applied the same principle to identify additional genes under purifying selection. As differences in functional impact scores only become statistically detectable when a minimal number of mutations are present, the screen was limited to 1,187 expressed genes containing at least 10 mutations in both the HeZD and the CNN group (see Methods). Included genes were therefore located in genomic regions with elevated mutation rates (median 5.5 versus 2.0 mutations per Mb per sample for screened and non-screened genes respectively). Of these 1,187 evaluated loci, seventy-six and twenty-four showed reduced CADD scores in HeZD compared to CNN tumors at an unadjusted *P*-value threshold of 0.05 and false discovery rate (FDR) of 50%, respectively (Fig 3A and 3B, S1 Table). Similar numbers of genes were detected using the other functional impact scores (S1 Table). As expected, highly ranked genes typically showed dN/dS ratios below 1 in HeZD tumors, consistent with avoidance of non-silent mutations and hence purifying selection (Fig 3C). Genes at the opposite end of the rank scale, which instead had elevated functional impact in HeZD compared to CNN tumors, typically showed dN/dS ratios above 1. The latter is consistent with loss of heterozygosity events where function-altering mutations have been positively selected for in the remaining copy of the HeZD gene, as confirmed by strong enrichment of tumor suppressors among these genes (Fig 3D, S3 Fig).

The highest-ranking gene was *INTS10* on chromosome 8p *(P =* 1.6*10^−4^, FDR = 0.19; Fig 3A, 3E and 3F, S1 Table*)*, hemizygously deleted in 29% of samples and, interestingly, a known interaction partner of *POLR2A* [21]. *POLR2A*, on chromosome 17p, was found on rank 14 (*P* = 5.8*10^−3^, FDR = 0.47). Several other top-ranking genes were similarly related to transcription or RNA processing, including bromodomain proteins *BRPF1* and *BRD1* (*P* = 3.5*10^−4^, FDR = 0.21 and *P* = 2.6*10^−3^, FDR = 0.39, respectively), and *PRPF8* (*P* = 6.4*10^−3^, FDR = 0.47), a key component of the spliceosome [22]. Notably, like *POLR2A*, *PRPF8* co-localizes with *TP53* (89.3% of *TP53* deletions had concomitant deletion of *PRPF8*). A gene set enrichment analysis (GSEA) of high-ranking genes (76 genes with unadjusted *P*≤0.05) confirmed strong enrichment of RNA-related pathways including RNA processing (FDR = 2.8*10^−3^), translation (FDR = 0.13) and RNA splicing (FDR = 0.13), supporting that the integrity of RNA processing is essential for cancer cell viability (Fig 3G, S2 Table). To further evaluate whether RNA-related pathways are subject to purifying selection, we pooled together mutation data from all genes belonging to the same GO pathway and confirmed that CADD impact scores were significantly reduced in HeZD compared to CNN samples for similar RNA-related pathways (S3 Table).

Similar to *POLR2A*, normalized CADD scores confirmed that the cancer-specific mutational signatures were not confounding these results (S1 Table; top 5 genes shown in Fig 3F). These scores also indicated that the effect was mainly attributed to purifying selection (negative scores) in the HeZD group rather than positive selection (positive scores) in the CNN group (Fig 3H and S1 Table). This was further supported by the observation that, for the top ranking 76 genes, CADD scores in amplified samples were more similar to CNN than HeZD samples (S4 Fig and S1 Table). Additionally, except for *ZNF292* (at rank 69, *P* = 0.044), all HeZD events were attributed to broad, rather than focal, deletions, consistent with passenger effects rather than driver roles for these deletions.

Copy number data inferred from whole genome sequencing was available for a subset of 1,065 samples (13.5%) and correlated strongly with the original SNP6-based data (Spearman rank coefficient 0.79, *P* = 0; S5 Fig). Repeating the analysis based on this data confirmed the trend towards purifying selection in the HeZD state, although the statistical strength was limited by the low number of samples (S5 Fig and S1 Table).

### Genes under purifying selection belong to protein complexes

Analysis of human population copy number variants has suggested that members of protein complexes tend to be under elevated purifying selection to avoid dosage changes [23]. We found that 12/24 (50%) of genes at 50% FDR were part of experimentally determined protein complexes as described in the CORUM database [24], compared to 24% for genes showing no evidence of purifying selection (*P* = 0.0063, Fisher’s exact test; Fig 3I). Several individual CORUM protein complexes were enriched among the high-ranking (*P*≤0.05) genes, again predominantly RNA-related and including spliceosome, integrator, and mRNA decay complexes (S2 Table). CADD impact scores were also significantly reduced in HeZD compared to CNN samples for these complexes after pooling all mutation data from genes active in the same protein complex (S3 Table).

Taken together, our results support that purifying selection acts to preserve the function of protein complex genes involved in RNA processing events (Fig 3J).

### Genes under purifying selection are essential for cancer cells

**Fig 4:**
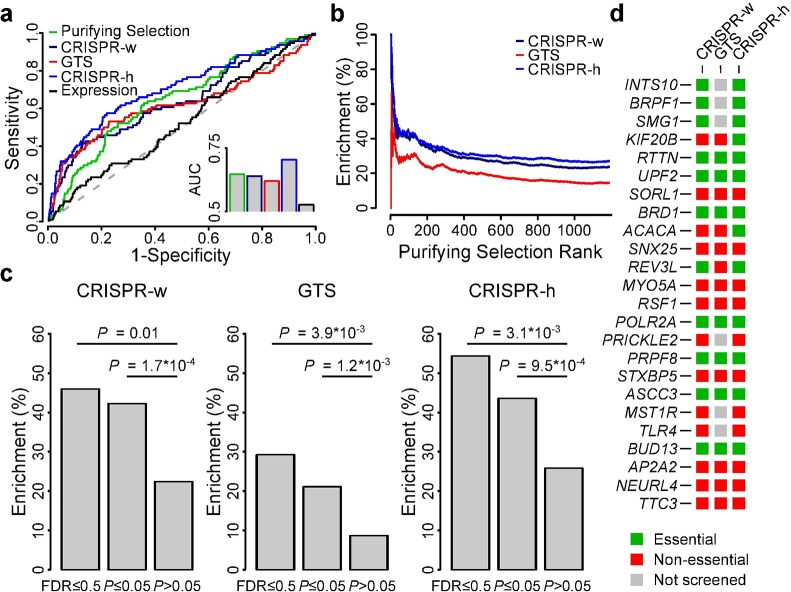
Genes subject to purifying selection are identified by orthogonal methods in cell lines. Comparison of genes showing signals of purifying selection with essential genes identified by CRISPR/Cas9 and gene-trap based methods in cell lines. **(a)** Receiver Operating Characteristic (ROC) curves using *S*. *Cerevisiae* essential gene homologs as a benchmark dataset. Area under the curve (AUC) values are compared in the inset. **(b)** Enrichment results. Curves show the cumulative proportion of experimentally-derived essential genes in cell lines as a function of purifying selection-based gene rank. **(c)** Bar plots show the proportion of genes identified to be under purifying selection (at 50% FDR or *P*≤0.05) that were retrieved by orthogonal methods. **(d)** Overview of the 24 genes identified at 50% FDR in this study and their identification by orthogonal methods.

We next aimed to compare our findings to orthogonal data from recent CRISPR/Cas9 and gene-trap-based screens for gene essentiality in cancer cell lines [9,25]. Human orthologs to essential genes in the yeast *S*. *Cerevisiae* [26] were used as a benchmark set, and we found that the genes we identified based on purifying selection criteria were comparable to these approaches in terms of prediction accuracy (area under the ROC curve metric, Fig 4A). Furthermore, there was a significant enrichment of genes identified using these orthogonal methods among top-ranking genes from our screen (Fig 4B and 4C). Several genes were verified as essential by multiple independent studies (e.g. *INTS10*, *BRPF1*, *SMG1*, *RTTN*, *UPF2*, *BRD1*, *POLR2A*, *ASCC3*, *PRPF8*, *BUD13*) (Fig 4D). These results further support that many genes that show patterns of reduced mutation impact in HeZD compared to CNN samples are indeed essential genes under purifying selection to maintain their function in tumors.

It has been shown that some protein complexes are comprised mostly of essential genes (referred to as modular essentiality) [27]. Using the criteria described by Hart et al. [27] (i.e. essential complexes contain at least 70% essential genes) and defining gene essentiality based on the experimental CRISPR/Cas9 screens mentioned above, we found that 41% of all protein complexes considered above were essential, and that the gene set enrichment results on protein complexes could mainly be attributed to this essential subset (S2 Table).

Finally, the genes we identified under purifying selection were also weakly enriched for loss-of-function intolerant genes that were recently described by the Exome Aggregation Consortium [28] (S6 Fig).

### Reduced promoter hypermethylation events in genes under purifying selection

In addition to somatic mutations, genes can get inactivated by other genomic events like homozygous deletions (HoZD) or promoter methylations. As genes subject to purifying selection are not expected to undergo these events, we analyzed our identified genes for such alterations.

**Fig 5:**
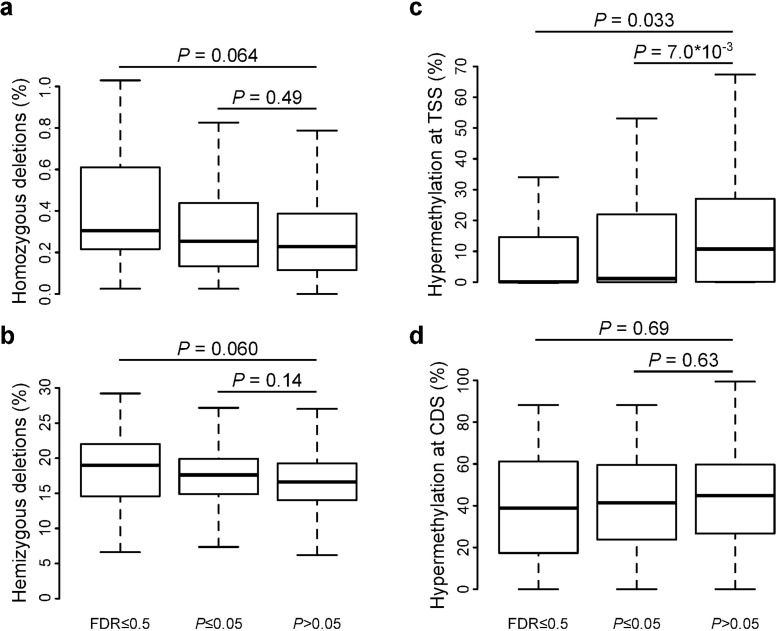
Deletion and promoter hypermethylation events in genes subject to purifying selection. Boxplots show for both sets of genes with signals of purifying selection (at 50% FDR and *P*≤0.05) and the other screened genes (*P*>0.05) the proportion of samples containing homozygous deletions **(a),** hemizygous deletions **(b)**, transcription start site (TSS) hypermethylations **(c)** and gene CDS hypermethylations **(d)**.

Unexpectedly, we found no difference between the proportion of samples that contained HoZD in the 76 top-ranked genes from our purifying selection screen as compared to the other genes screened for (0.25% versus 0.23%, *P* = 0.49, two-sided Wilcoxon rank sum test; Fig 5A). In fact, the 24 highest ranked genes even had a higher proportion of HoZD (0.30%, *P* = 0.064). As we also found a higher proportion of the much more frequently occurring hemizygous deletions for these genes (19.0% versus 16.5%, *P* = 0.060; Fig 5B), we hypothesize that this apparent lack of depletion of HoZD might be due to segmentation value variability resulting from tumor sample impurities (i.e. primary tumors are admixtures with normal human cells) and resulting misclassifications of HeZD into HoZD. As cancer cell lines do not suffer from this effect, and have been suggested to be superior to primary tumors for identification of HoZD [29], we extended the analysis of HoZD to an independent cell line dataset from the Cosmic Cell Lines Project [30]. Here, we could demonstrate a reduction in HoZD for the top-ranked genes compared to genes without any signals of purifying selection (*P* = 0.039 and P = 0.067 for the 24 and 76 highest ranked genes respectively), as would be expected for genes under purifying selection; S7 Fig)

Additionally, CpG promoter hypermethylations were found to occur less frequently in the 76 highest ranked genes identified to be under purifying selection (1.1% versus 10.7%, *P* = 0.0070, two-sided Wilcoxon rank sum test; Fig 5C). This effect was found to be restricted to promoter regions and not the gene body (Fig 5D), excluding a confounding effect from copy number state.

Overall, these results confirm that genes under purifying selection not only avoid deleterious somatic mutations, but also other damaging genomic events.

## Discussion

In this study, we provide results suggesting that somatic mutations that occur in the remaining copy of HeZD essential genes are subject to purifying selection in cancer cells. While numerous studies and methods have been published that detect positive selection in tumor cells, mainly aiming to detect cancer driver genes, purifying selection is understudied in cancer, and it has been suggested that purifying selection is limited compared to germline evolution [6,7]. We suggest two main reasons for this lack of previous studies and results: 1) a lack of statistical power to detect purifying selection and 2) the haplosufficient nature of many essential genes in tumors.

The detection of positive and purifying selection implies identification of function-altering somatic mutations that occur more or less frequently in a given gene or genomic location than would be expected by random chance. In genes subject to positive selection, an expected elevation in mutation count is helpful in terms of statistical power. In the case of purifying selection, which essentially implies studying mutations that are not present anymore, obtaining enough observations to reach sufficient statistical power may require considerably larger cohorts. This is exemplified in large B-cell lymphoma by *BCL2*, a rare case where purifying selection has been described previously [31]. In this gene, purifying selection became detectable only because of a strongly elevated local somatic mutation frequency due to AID-mediated somatic hypermutation acting on this locus. For similar reasons, our screening was limited to genomic regions containing higher mutation rates. This restricted the analysis to 1,187 genes, which implies that the total number of genes under purifying selection is likely much larger than the set of 76 candidate genes we identified. The underestimation of the actual number of genes under purifying selection is reinforced by the fact that purifying selection in HeZD genes is expected to result in lower mutation rates due to both the loss of one mutable gene copy as well as the purifying selection-driven reduction in high impact mutations itself.

To increase the statistical power in this study, we pooled together somatic mutation and copy number data from 25 cancer types, encompassing more than 7,000 samples, which has become feasible only recently thanks to advances in sequencing technologies and large scale cancer genome initiatives like TCGA. While this has the potential to identify purifying selection in genes that are globally essential for cancer cells, the signals we identified remained statistically weak and no conclusions could be drawn at the level of individual cancer types. However, statistical power and hence the number of screenable genes will grow stronger as data continue to accumulate, which may allow identification of more genes under purifying selection, even in individual cancer types.

By normalizing the CADD functional impact score to the expected impact score in the absence of any selection pressure using a simulation approach, we excluded any bias resulting from differences in mutational signatures between cancers. This normalization also indicated that, for most identified genes, the difference in functional impact between HeZD and CNN samples was due to purifying selection in the HeZD state, rather than positive selection in the CNN state. For a minority of genes, positive selection in the CNN state could not be excluded. This is to be expected for haploinsufficient tumor suppressor genes, in which the functional loss of one copy leads to a fitness advantage [32]. The gene with the highest signal of positive selection in the CNN state was *CTCF*, a gene that has indeed been proposed as a haploinsufficient tumor suppressor gene [33].

Targeted therapies in cancer are traditionally focusing on driver genes and more specifically the inhibition of overactive oncogenes. An interesting alternative strategy is the inhibition of essential genes. However, our knowledge of essential genes in human cancer cells is limited. Several recent studies identified essential genes in human cancer cell lines using the CRISPR/Cas9 technique [9,25,34]. The overlap that was shown between our results and these orthogonal approaches supports the validity of the signals detected with our approach, and demonstrates that patterns of purifying selection have the potential of revealing gene essentiality in primary tumors. It is indeed important to realize that cell essentiality might strongly differ between cell lines and primary tumors, as the former lack interactions with the tumor microenvironment and the host immune system. In this context, it is interesting to note that *TLR4* and *MST1R*, both receptors of immune modulators, were found to show putative signals of purifying selection but were not identified by these gene essentiality screens.

Genes that were shown to be under purifying selection were remarkably enriched for RNA-related events (i.e. RNA processing, splicing and nonsense-mediated mRNA degradation). While RNA processes have been found to be essential in human cell lines before [9,11,34,35], this is the first study to demonstrate this in primary tumor samples. *INTS10*, the gene with the strongest signal of purifying selection, is a known interaction partner of *POLR2A*, which was used as to establish proof of concept in this study. *INTS10* is part of the integrator complex, a complex of proteins that is mainly involved in the transcription of small nuclear RNAs, but has also been linked to transcriptional pausing [21] and has not been linked to cancer previously. The importance of splicing events for tumor cell viability is in line with the enhanced cytotoxicity that has been observed when mRNA splicing was inhibited in cancer cells [36,37]. *PRPF8*, one of the key proteins in the spliceosome, is located on the same *TP53*-associated deletion as *POLR2A*. As *POLR2A* inhibition in these cells has been shown to have therapeutic potential previously [3], this raises the possibility that simultaneous inhibition of *PRPF8* could increase the therapeutic window in this potentially large group of patients. The other genes we identified to be under purifying selection were likewise hemizygously deleted in large proportions of samples, which suggests that a large fraction of tumors may be vulnerable to therapeutic inhibition of HeZD essential genes.

Previous screens in cell lines have indeed suggested that many hemizygous deletions sensitize cancer cells to inhibition [10,11], but it has not been possible yet to systematically screen for this special form of synthetic lethality in primary tumor samples. The approach proposed here promises to circumvent that limitation, by providing indirect evidence of vulnerabilities that result from widespread deletions that occur during tumor evolution. However, a major limitation could be narrow therapeutic windows resulting from the fact that most of the retrieved genes are involved in RNA-related processes that are crucial for normal cell viability.

## Conclusions

We have provided results supporting that, with the current amount of available somatic mutation data from tumors, and by considering HeZD genomic regions, purifying selection against damaging somatic mutations is becoming discernible in individual essential genes. Since these deletions occurred in up to 29% of all tumors, our approach may reveal frequently occurring cancer vulnerabilities that can be exploited by future therapies.

## Methods

### Somatic mutation data

Mutation annotation format (maf) files from 26 different cancer types were downloaded from The Cancer Genome Atlas (TCGA) data portal. Selection and processing of data was performed as suggested by Kandoth *et al*. [38]. Data from colon and rectal adenocarcinoma were concatenated. Mutation data that were annotated in hg18 were converted to hg19 using UCSC’s liftOver [39]. All duplicate lines, identified as mutations with a similar sample id and genomic location, were removed from the final dataset.

ANNOVAR [40] was used for mutation annotation, calculation of functional impact scores and extraction of information regarding coding DNA sequencing (CDS) positions. Mutation Assessor, SIFT and PolyPhen-2 scores were optimized for silent and truncating mutations using the criteria of Gonzalez-Perez and Lopez-Bigas [41]. A similar principle was applied for (phred-like) Combined Annotation-Dependent Depletion (CADD) scores in which silent and truncating mutations were set to values of 0 and 40 respectively.

### Copy number data

Segmented genomic copy number alteration (CNA) data (Affymetrix SNP6 platform, minus germline) were downloaded from the Broad Institute. GISTIC 2.0 was used to derive copy number status information for each gene and sample [42]. GISTIC 2.0 was run using parameter settings as performed by the Cancer Genome Atlas Network [43]. Focality of deletions was determined from GISTIC output and visual confirmation using the Integrative Genome Viewer [44].

### Gene expression data

Level 3 RNASeqV2 (RSEM normalized) mRNA expression data were downloaded from the TCGA data portal. Gene expression was evaluated for each cancer type. A gene was considered expressed when the median RSEM expression value in a cancer type was higher than 200. Only expressed genes were used for further analysis.

### Methylation data

Human methylation 450 platform data were downloaded from the TCGA data portal with the bioconductor package TCGAbiolinks [45]. The methylation probes were associated with gene transcription start site or CDS using annotation files provided by Illumina. Genes with beta-values > 0.7 were considered hypermethylated.

### Genome-wide screen for genes under purifying selection

To avoid any bias in the detection of purifying selection due to the clustering of (silent) mutations at similar genomic locations caused by positive selection as has been described for different cancer driver genes [46,47], we removed all clustering mutations using a binomial approach as described previously [48]. Using this approach, mutations were defined to be clustered when there were more mutations at the same genomic positions than could be expected when mutations would occur randomly in the gene (at *P*≤0.01) given the total number of mutations for that gene and the gene length.

As negative selection is only expected to occur in genes that are expressed by a tumor cell, we further restricted the analysis to mutations occurring in expressed genes (as defined above). Data from somatic mutations hitting non-expressed genes were used for negative control purposes.

Genes under purifying selection were defined as those that had a lower CADD functional impact score in samples harboring a hemizygous deletion as compared to copy number neutral samples (using a one-sided Wilcoxon rank sum test). Because these differences in functional impact scores can only be determined when a minimal number of mutations are present in both groups, the analysis was limited to genes containing at least 10 mutations in both the hemizygous deletion and the copy number neutral group. These filter criteria yielded a set of 1,187 genes that could be screened for signals of purifying selection. FDR values were calculated using the Benjamini Hochberg method [49]. Purifying selection ranks were based on increasing p-values (i.e. the highest ranked gene is the gene with the most significant difference between hemizygously deleted and copy number neutral tumors). Correlations with dN/dS values were determined using Spearman’s rank correlation coefficient.

### Mutational signatures

The 96 mutational substitution classes as defined by Alexandrov *et al*. [19] were determined for all mutations. These substitution classes are defined by the 96 possible combinations of the 6 base substitutions referred to by the pyrimidine of the mutated base pair (i.e. C>A, C>G, C>T, T>A, T>C and T>G), the 4 upstream and the 4 downstream base pairs.

### Calculations of dN/dS

The metric dN/dS is the ratio of non-silent to silent mutations per site [13,14]. For each gene, somatic point mutations annotated by ANNOVAR as *synonymous SNV* were classified as silent mutations (s). All other annotated point mutations were classified as non-silent (n). The number of non-silent and silent mutation sites was calculated by simulating all three possible substitutions for each CDS position in a specific gene (i.e. each nucleotide can theoretically be mutated in three other nucleotides). Because the probability of any somatic mutation hitting a particular site is defined by the underlying mutational processes and hence its mutational signature, we determined the proportions of each substitution class (96-class model) for each gene and copy number state (HeZD and CNN) and used these as prior probabilities to determine the theoretical number of silent (S) and non-silent (N) mutation sites. Therefore, we used a sampling approach with a mutation probability determined by its specific substitution subclass. Sampling was done 1 million times to minimize variability. dN/dS was then calculated as the ratio of n/N over s/S. Fisher’s exact test (one-sided) was used to check whether dN/dS ratios were lower than 1 and hence indicative of purifying selection.

### Normalized CADD score calculations

To determine the expected functional impact (CADD) score in the absence of any selection pressure, we calculated the scores for each possible point mutation in a gene using ANNOVAR and used the same 96-class mutational signature-based sampling approach as described higher to get the simulated functional impact score. The normalized CADD score was then calculated by normalizing the difference between each observed CADD score and the median of the simulated score to the maximal difference for each cancer (as shown in S2 Fig). This way, normalized CADD scores between 0 and 1 indicate positive selection, while values between 0 and -1 indicate purifying selection. Deviations from 0 were evaluated for each copy number state (HeZD and CNN) using a one sample one-sided Wilcoxon rank sum test.

### Gene set enrichment analysis

A gene set enrichment analysis (GSEA) was performed to determine the pathways and protein complexes in which the retrieved genes were active. GO biological process gene sets were downloaded from the Molecular Signatures Database v5.0 [50] and CORUM was used as a resource for protein complexes [24]. Only GO processes or CORUM complexes that contained at least 2 genes under analysis were used for the GSEA. Pathway or protein complex enrichment was determined using one-sided Fisher’s exact test at 25% FDR.

### Comparison with genes retrieved by orthogonal methods in cell lines

The purifying selection results were compared with the results from two recent orthogonal methods to identify essential genes in human cancer cell lines: two CRISPR/Cas9 methods (referred to as CRISPR-w [9] and CRISPR-h [25]) and one gene-trap study [9] (referred to as GTS). To evaluate and compare the accuracy to predict cell essentiality using signals of purifying selection and these orthogonal methods, a receiver operating characteristic (ROC) analysis was performed using the human homolog genes of the *S*. *cerevisiae* essential genes as a benchmark [26]. Only genes present in all compared datasets were used for analysis. The datasets were ranked based on increasing p-values (purifying selection), maximal CRISPR scores in the 4 analyzed cell lines (CRISPR-w), maximal Bayes Factor in the 5 analyzed cell lines (CRISPR-h) and the gene-trap score from the gene-trap study. Gene expression values were used as a control dataset.

The enrichment of essential genes identified by these orthogonal methods for the purifying selections results was evaluated using Fisher’s exact test. Genes were defined essential when identified as essential in at least one cell line in the corresponding CRISPR screen. The gene-trap approach can only identify essential genes in haploid regions, and diploid genes (located on chromosome 8 and part of chromosome 15 in the KBM7 cell line) were therefore excluded.

### Statistical analysis

The R statistical package was used for all data processing and statistical analysis. Details on statistical tests used are reported in the respective sections. The Manhattan plot and *S*. *Cerevisiae* essential gene homologs were determined using the Bioconductor package [51].

## Supporting Information

S1 Fig Cancer type-specific differences in *POLR2A* functional impact scores. The functional impact of all observed mutations in *POLR2A* was predicted using the CADD score for both copy number groups and each cancer type. p-values are indicated above the plots for those cancers that contain mutations in both groups. Nine out of eleven analyzable cancer types (as indicated below each plot) contained a lower CADD score in HeZD samples, although this never reached statistical significance. Mutations in HeZD and CNN samples are indicated by asterisks and dots respectively. Horizontal lines on plots indicate median values.
(PDF)

S2 Fig Normalized CADD scores correct for mutational signature differences between cancers. **(a)** Pie charts show the proportion of the 25 different cancer types in samples that are CNN for *POLR2A* or contain a HeZD in this gene. **(b)** Mutational signature examples of metastatic melanoma (SKCM) and breast cancer (BRCA). A signature is determined by the contribution of 96 mutation classes, i.e. the combination of 6 substitutions (shown in colors), 4 up- and 4 downstream base pairs, as indicated. **(c)** CADD scores were simulated for *POLR2A* using the cancer-specific mutational signature as prior mutation probabilities (see Methods). Median simulated CADD scores are shown for each cancer. **(d)** Normalization approach. Each observed CADD score (red dots, CADD_obs_) is normalized to the median simulated score (blue line, CADD_sim_). **(e)** CADD normalization exemplified for *POLR2A*.
(PDF)

S3 Fig Positive selection of mutations occurring in hemizygously deleted tumor suppressor genes. A genome-wide screen was performed on 1,187 genes to detect differences in the CADD scores between CNN and HeZD genes using a two-sided Wilcoxon rank sum test. **(a)** qq-plots show the negative effect (CADD score HeZD<CNN) on the upper left side and the positive effect (CADD score HeZD>CNN) on the bottom right side. **(b)** Copy number-specific CADD (left) and normalized CADD (right) scores from the observed mutations in *RB1*. **(c)** Proportion of Cancer Gene Census (CGC) genes for genes with (+) or without (-) signals of positive selection in the HeZD state (defined as FDR≤0.25). **(d)** Proportion of CGC genes known to be dominant and recessive for genes with or without signals of positive selection in the HeZD state. As classical (two-hit) tumor suppressor genes are expected to operate in a recessive way, these differences in proportions clearly suggest an enrichment of tumor suppressor rather than oncogenes.
(PDF)

S4 Fig CADD scores of amplified genes that were identified to be under purifying selection. For each gene that was identified to be under purifying selection (76 top ranked genes), the median CADD score was determined for HeZD, CNN and amplified (Amp) samples. **(a)** Correlation between median CADD scores of Amp versus HeZD (left) and CNN (right) genes. Spearman correlation coefficients and p-values are indicated on top of the plots. **(b)** Boxplots of median CADD scores for the 3 copy number states. Results from individual gene analyses are indicated in S1 Table.
(PDF)

S5 Fig CADD functional impact score differences after inferring copy number state from whole genome sequencing data. **(a)** Cancer types and number of samples for which WGS segmentation data were available. **(b)** Correlation between gene-based segmentation values derived from SNP6 arrays as a function of values derived from WGS. Each dot represents a segmentation value of one gene in one sample. For visualization purposes the points shown are limited to a random set of 10,000 points. Spearman’s correlation coefficient is shown on top. **(c)** Copy number-related CADD scores from the observed mutations in the top-5 ranked genes from the main analysis. Horizontal lines on plots indicate median values. WGS-based copy number segmentation data were downloaded from TCGA and analyzed using GISTIC 2.0. Results from individual gene analyses are indicated in S1 Table.
(PDF)

S6 Fig Enrichment results for loss-of-function intolerant genes. Bar plot shows the proportion of genes identified to be under purifying selection (at 50% FDR and *P*≤0.05 respectively) and the other screened genes (*P*>0.05) that were previously described as intolerant to loss-of-function variants by the EXaC consortium [28].
(PDF)

S7 Fig Cell line deletions of genes identified to be under purifying selection. Boxplots show for both sets of genes under purifying selection (at 50% FDR and *P*≤0.05 respectively) and the other screened genes (*P*>0.05) the proportion of cell lines containing hemizygous **(a)** or homozygous **(b)** deletions. **(c)** Bar plot shows the proportion of genes containing at least 1 HoZD. P-values were calculated using two-sided Wilcoxon rank sum test (panels **a** and **b**) or Fisher’s exact test (panel **c**). Copy number data were downloaded from the Cosmic Cell Lines Project [30].
(PDF)

S1 Table Purifying selection results on a predefined 1,187 gene set. Genes were ranked based on increasing p-values for the CADD scores (*p_CADD* column in first tab). p-values were calculated using a one-sided Wilcoxon rank sum test to identify genes with lower functional impact scores in the HeZD compared to the CNN state. Different tabs show the results from the main analysis (1), normalized CADD scores (2), analysis focusing on amplified genes (3) and after classifying copy number changes using WGS data (4).
(XLSX)

S2 Table Gene set enrichment analysis. Enrichments are calculated using Fisher’s exact test on genes showing any sign of purifying selection (76 gene set). Pathways are ranked based in increasing enrichment p-values. Tabs show results of enrichment for genes active in GO biological processes (1), CORUM protein complexes (2) and CORUM essential protein complexes (3). Essential protein complexes were defined as protein complexes that contain at least 70% essential genes, in which gene essentiality was determined based on the CRISPR screens by Hart et al. and Wang et al. [9,25].
(XLSX)

S3 Table Pooled purifying selection analysis. Mutation data from genes active in the same GO biological process (first tab) or CORUM protein complex (second tab) were pooled and both copy number states were analyzed for differences in CADD functional impact scores, similar to the main analysis. Pathways/Complexes are ranked based on increasing p-values.
(XLSX)
