# Supplementary figures and images for: Somatic Mutation Patterns in Hemizygous Genomic Regions Unveil Purifying Selection during Tumor Evolution
Source: PLoS Genet. 2016 Dec 27;12(12):e1006506. doi: 10.1371/journal.pgen.1006506 (PMC5189942; doi:10.1371/journal.pgen.1006506)

Supplementary Figure 1

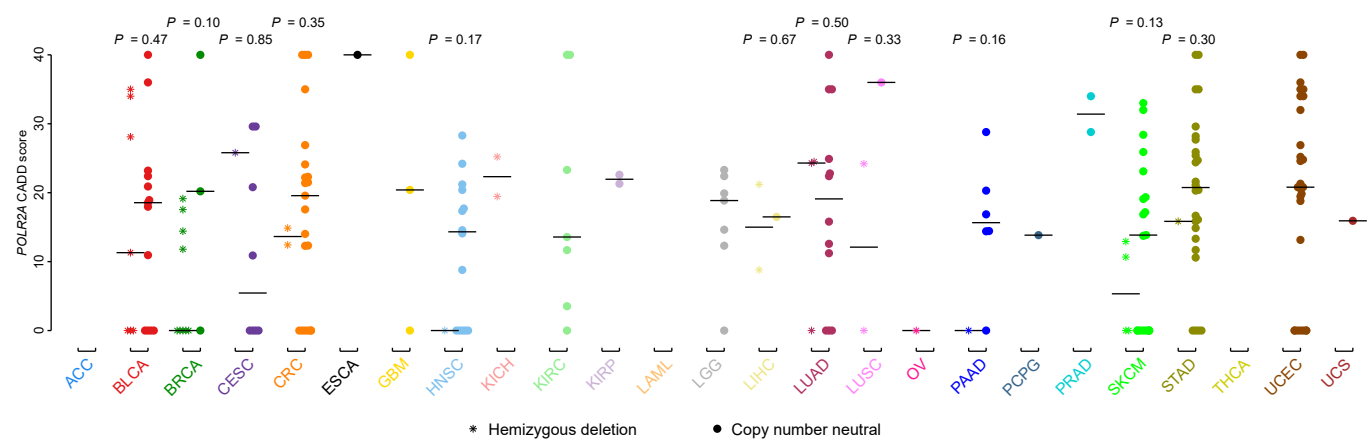

Supplement: S1 Fig — The functional impact of all observed mutations in POLR2A was predicted using the CADD score for both copy number groups and each cancer type. p-values are indicated above the plots for those cancers that contain mutations in both groups. Nine out of eleven analyzable cancer types (as indicated below each plot) contained a lower CADD score in HeZD samples, although this never reached statistical significance. Mutations in HeZD and CNN samples are indicated by asterisks and dots respectively. Horizontal lines on plots indicate median values. (PDF) [file pgen.1006506.s001.pdf]

# Supplementary Figure 2

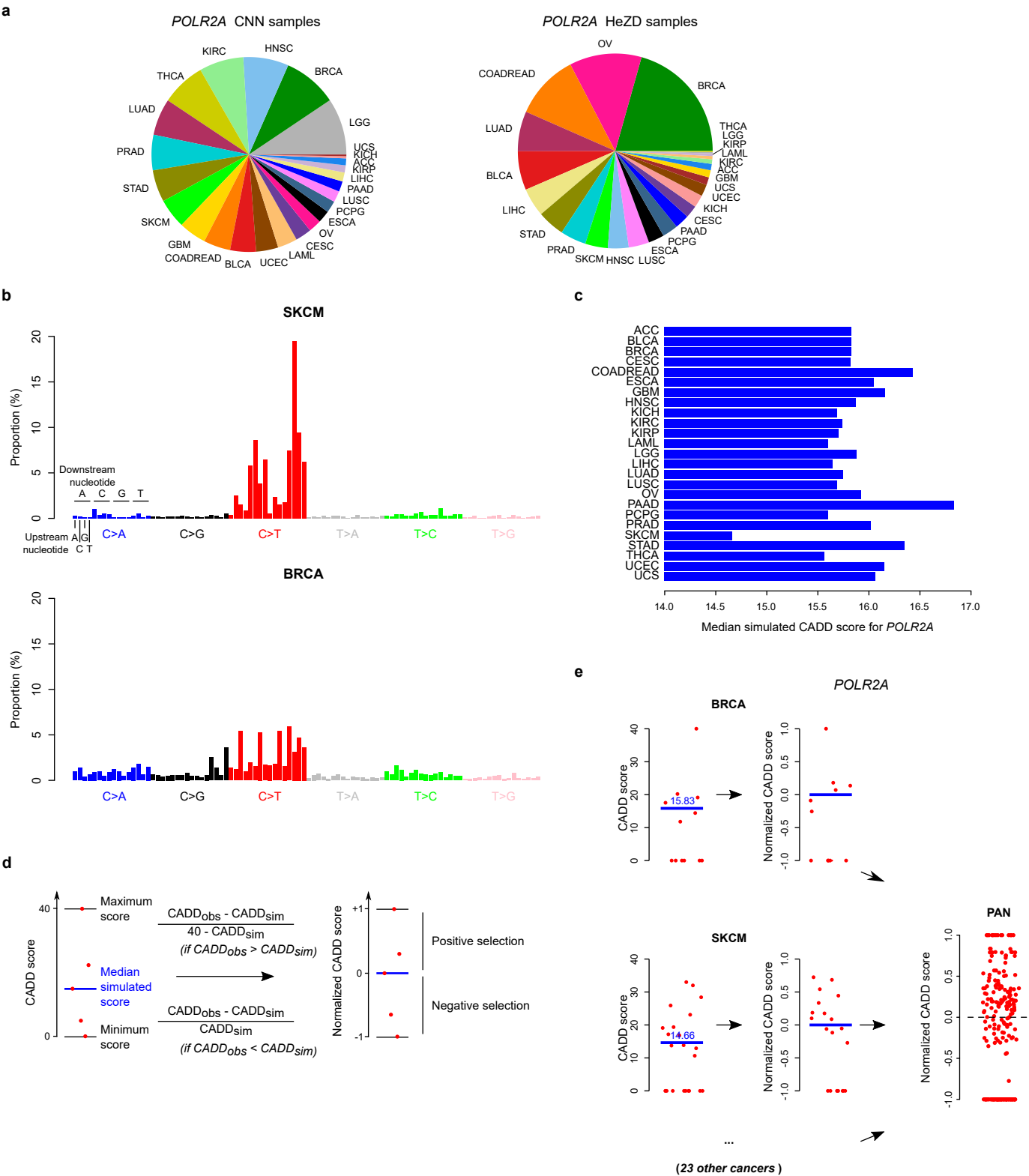

Supplement: S2 Fig — (a) Pie charts show the proportion of the 25 different cancer types in samples that are CNN for POLR2A or contain a HeZD in this gene. (b) Mutational signature examples of metastatic melanoma (SKCM) and breast cancer (BRCA). A signature is determined by the contribution of 96 mutation classes, i.e. the combination of 6 substitutions (shown in colors), 4 up- and 4 downstream base pairs, as indicated. (c) CADD scores were simulated for POLR2A using the cancer-specific mutational signature as prior mutation probabilities (see Methods). Median simulated CADD scores are shown for each cancer. (d) Normalization approach. Each observed CADD score (red dots, CADDobs) is normalized to the median simulated score (blue line, CADDsim). (e) CADD normalization exemplified for POLR2A. (PDF) [file pgen.1006506.s002.pdf]

Supplementary Figure 3

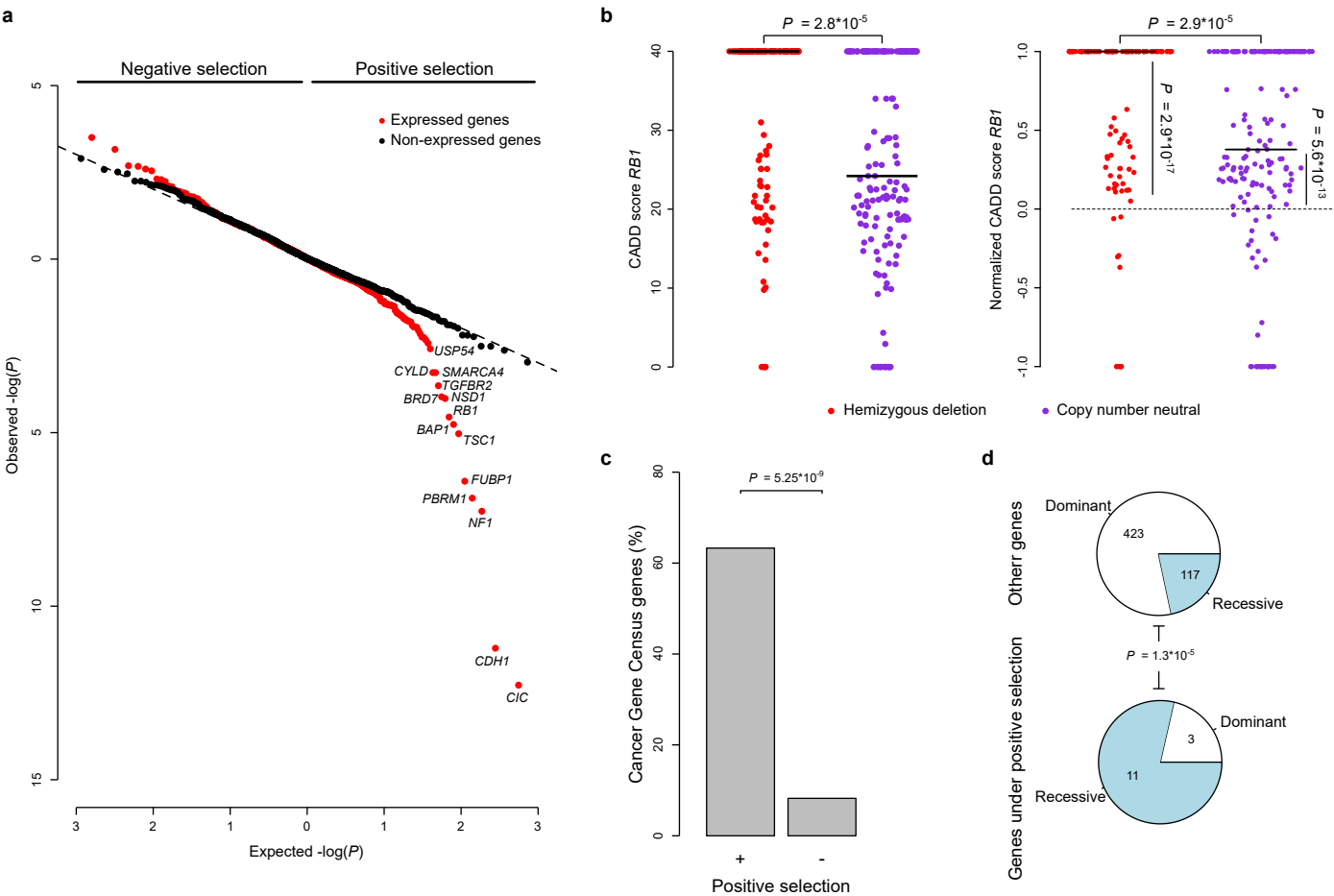

Supplement: S3 Fig — A genome-wide screen was performed on 1,187 genes to detect differences in the CADD scores between CNN and HeZD genes using a two-sided Wilcoxon rank sum test. (a) qq-plots show the negative effect (CADD score HeZD<CNN) on the upper left side and the positive effect (CADD score HeZD>CNN) on the bottom right side. (b) Copy number-specific CADD (left) and normalized CADD (right) scores from the observed mutations in RB1. (c) Proportion of Cancer Gene Census (CGC) genes for genes with (+) or without (-) signals of positive selection in the HeZD state (defined as FDR≤0.25). (d) Proportion of CGC genes known to be dominant and recessive for genes with or without signals of positive selection in the HeZD state. As classical (two-hit) tumor suppressor genes are expected to operate in a recessive way, these differences in proportions clearly suggest an enrichment of tumor suppressor rather than oncogenes. (PDF) [file pgen.1006506.s003.pdf]

Supplementary Figure 4

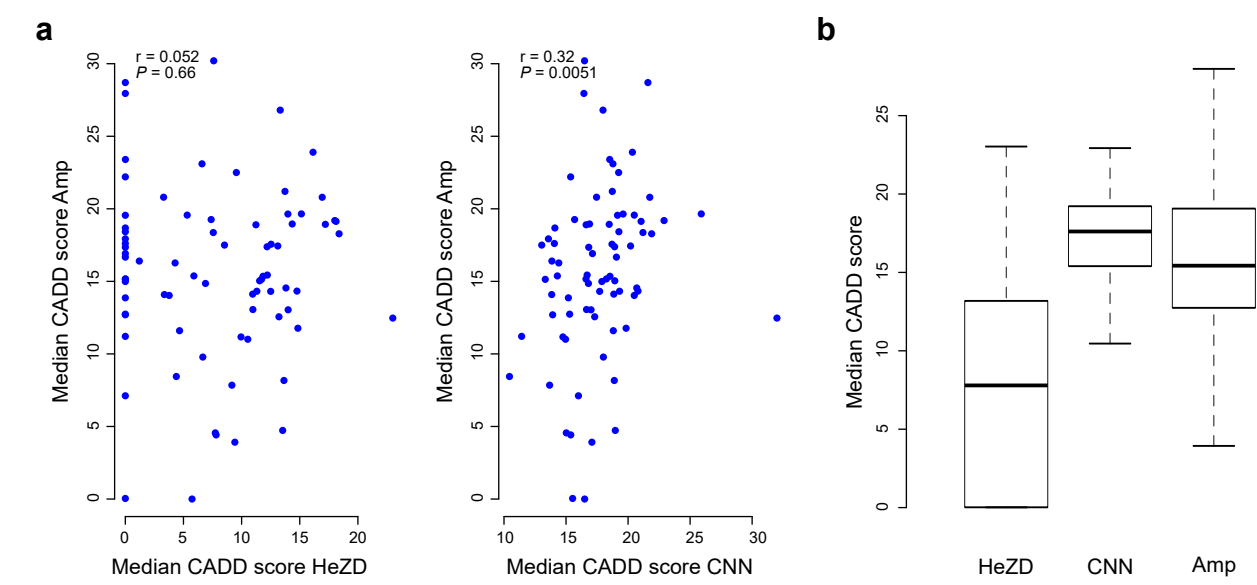

Supplement: S4 Fig — For each gene that was identified to be under purifying selection (76 top ranked genes), the median CADD score was determined for HeZD, CNN and amplified (Amp) samples. (a) Correlation between median CADD scores of Amp versus HeZD (left) and CNN (right) genes. Spearman correlation coefficients and p-values are indicated on top of the plots. (b) Boxplots of median CADD scores for the 3 copy number states. Results from individual gene analyses are indicated in S1 Table. (PDF) [file pgen.1006506.s004.pdf]

Supplementary Figure 5

a

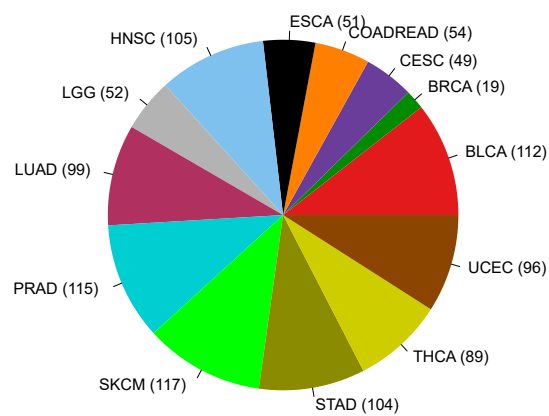

b

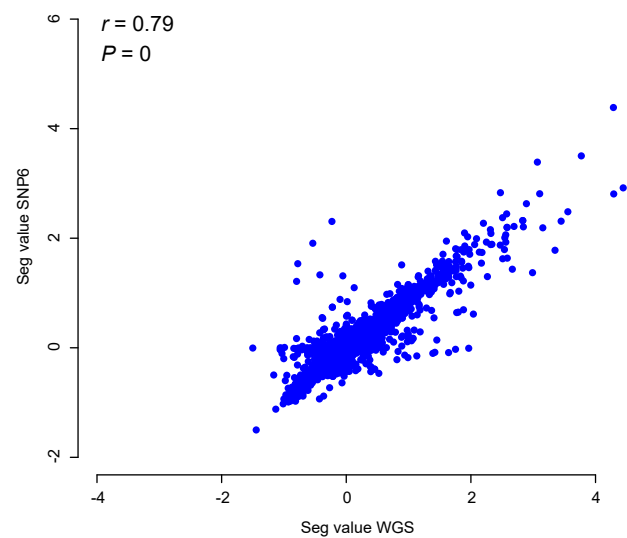

c

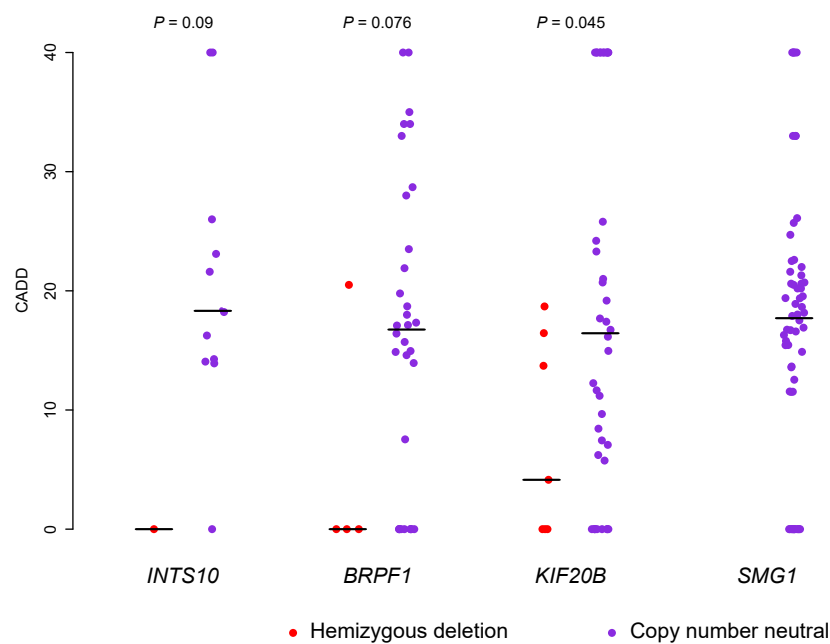

Supplement: S5 Fig — (a) Cancer types and number of samples for which WGS segmentation data were available. (b) Correlation between gene-based segmentation values derived from SNP6 arrays as a function of values derived from WGS. Each dot represents a segmentation value of one gene in one sample. For visualization purposes the points shown are limited to a random set of 10,000 points. Spearman’s correlation coefficient is shown on top. (c) Copy number-related CADD scores from the observed mutations in the top-5 ranked genes from the main analysis. Horizontal lines on plots indicate median values. WGS-based copy number segmentation data were downloaded from TCGA and analyzed using GISTIC 2.0. Results from individual gene analyses are indicated in S1 Table. (PDF) [file pgen.1006506.s005.pdf]

Supplementary Figure 6

EXaC loss-of-function intolerant genes

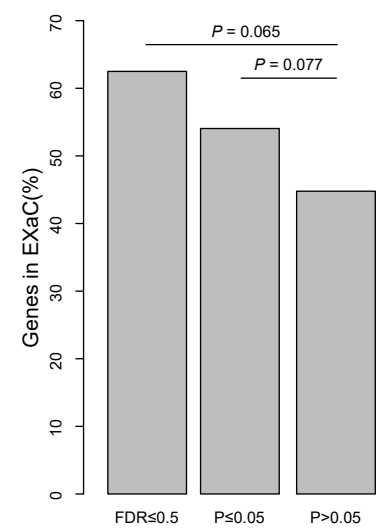

Supplement: S6 Fig — Bar plot shows the proportion of genes identified to be under purifying selection (at 50% FDR and P≤0.05 respectively) and the other screened genes (P>0.05) that were previously described as intolerant to loss-of-function variants by the EXaC consortium [28]. (PDF) [file pgen.1006506.s006.pdf]

Supplementary Figure 7

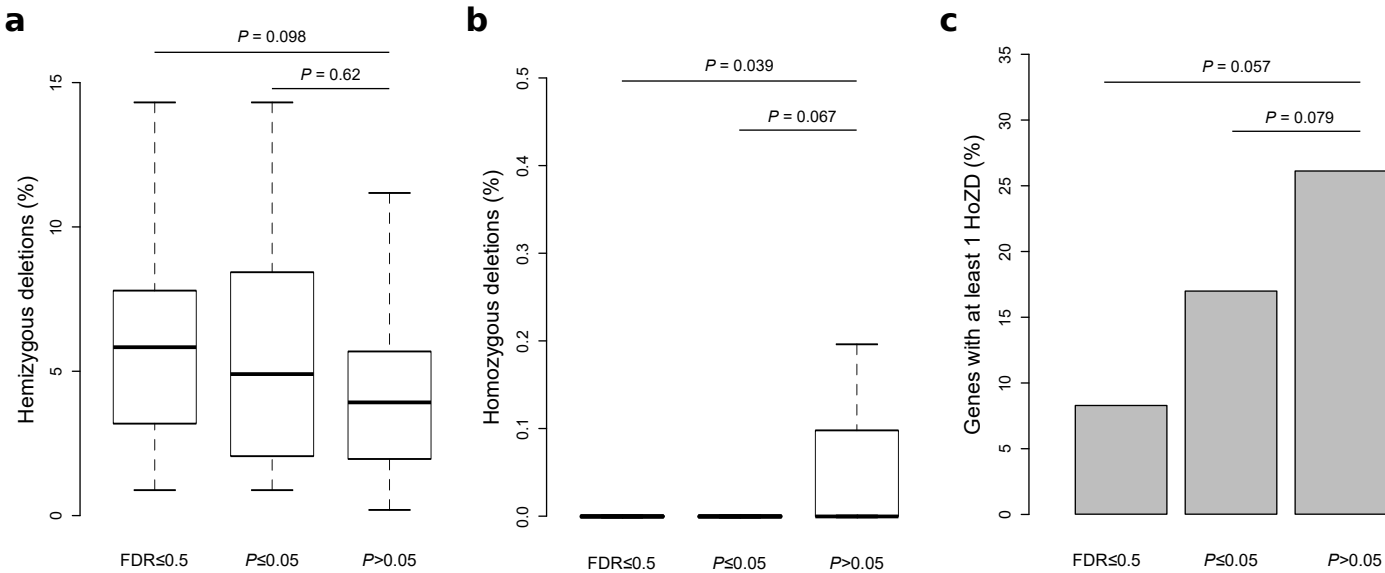

Supplement: S7 Fig — Boxplots show for both sets of genes under purifying selection (at 50% FDR and P≤0.05 respectively) and the other screened genes (P>0.05) the proportion of cell lines containing hemizygous (a) or homozygous (b) deletions. (c) Bar plot shows the proportion of genes containing at least 1 HoZD. P-values were calculated using two-sided Wilcoxon rank sum test (panels a and b) or Fisher’s exact test (panel c). Copy number data were downloaded from the Cosmic Cell Lines Project [30]. (PDF) [file pgen.1006506.s007.pdf]
